# Nighttime Image Dehazing by Render

**DOI:** 10.3390/jimaging9080153

**Published:** 2023-07-28

**Authors:** Zheyan Jin, Huajun Feng, Zhihai Xu, Yueting Chen

**Affiliations:** State Key Laboratory of Extreme Photonics and Instrumentation, Zhejiang University, Hangzhou 310027, China; 11930051@zju.edu.cn (Z.J.); fenghj@zju.edu.cn (H.F.); xuzh@zju.edu.cn (Z.X.)

**Keywords:** image dehaze, image restoration, data generation

## Abstract

Nighttime image dehazing presents unique challenges due to the unevenly distributed haze caused by the color change of artificial light sources. This results in multiple interferences, including atmospheric light, glow, and direct light, which make the complex scattering haze interference difficult to accurately distinguish and remove. Additionally, obtaining pairs of high-definition data for fog removal at night is a difficult task. These challenges make nighttime image dehazing a particularly challenging problem to solve. To address these challenges, we introduced the haze scattering formula to more accurately express the haze in three-dimensional space. We also proposed a novel data synthesis method using the latest CG textures and lumen lighting technology to build scenes where various hazes can be seen clearly through ray tracing. We converted the complex 3D scattering relationship transformation into a 2D image dataset to better learn the mapping from 3D haze to 2D haze. Additionally, we improved the existing neural network and established a night haze intensity evaluation label based on the idea of optical PSF. This allowed us to adjust the haze intensity of the rendered dataset according to the intensity of the real haze image and improve the accuracy of dehazing. Our experiments showed that our data construction and network improvement achieved better visual effects, objective indicators, and calculation speed.

## 1. Introduction

Daytime lighting is mainly affected by uniform atmospheric light, while nighttime lighting is primarily composed of artificial light sources, such as street lights and neon lights. These light sources are located at different positions and angles, have limited illumination ranges, and emit multiple colors, which can result in low visibility, uneven illumination, and color distortion in nighttime hazy images. Furthermore, the factors that affect fog absorption and scattering are complex, and there is a lack of reliable physical formulas and prior knowledge for nighttime haze. As a result, defogging nighttime images can be challenging. In practical nighttime scenarios, the performance of other computer vision tasks can be severely degraded, and the absence of reliable nighttime defogging algorithms can lead to failures in nighttime security monitoring and autonomous driving, and even traffic accidents. Removing non-uniform fog while maintaining color consistency in nighttime scenes is therefore a challenging and crucial task.

One approach to address the challenges of nighttime image dehazing is to estimate the extent and intensity of the haze illuminated by light sources through a network. However, the unstable range and intensity of lighting haze often result in prior conditions that differ significantly from real-world scenarios. Another approach to nighttime image dehazing involves constructing the data first and then training the network. This method involves applying different haze variations to images of objects with varying distances and textures on a two-dimensional (2D) image. However, this approach does not account for the illumination of the haze itself by the light source, resulting in suboptimal handling of the glow problem.

**Figure 1:**
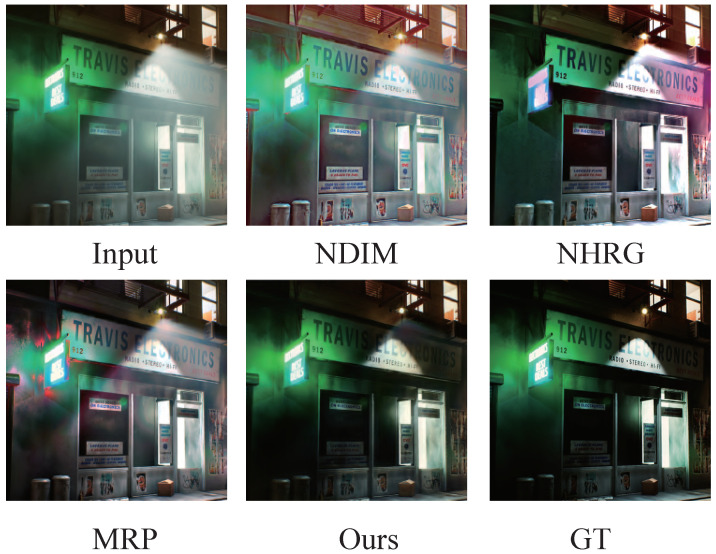
Comparison of nighttime dehazing results. Our method outperforms several state-of-the-art algorithms, including NDIM [1], NHRG [2], MRP [3], and OSFD [4], in effectively addressing fog caused by directional light sources, while maintaining color consistency. The data generated using our method ensure reliable scene details and realistic fog effects, thereby providing a more accurate representation of real-world scenarios.

To address these limitations, we propose a rendering engine-based method for generating nighttime dehazed image data that incorporates complex lighting and haze calculations in three-dimensional (3D) space. The 3D illumination and haze features are then remapped back to two-dimensional (2D) space by a virtual camera. We treat the interference of haze on light as the infection of point light sources by optical media and propose a point spread function for haze images that is similar to the light point spread function. Finally, we enhance the existing network architecture to enable better learning of the mapping from 3D engines to 2D datasets. In Figure 1, the results demonstrate that our proposed rendering data, haze point spread function detection, and improved network architecture contribute to effective improvements in nighttime image dehazing.

The contributions of our work are as follows:

- We convert the nighttime haze, which is challenging to accurately handle in two-dimensional space, to three-dimensional space. We propose a method for accurately describing nighttime haze using a three-dimensional scattering formula. Through the derivation of radiation transfer equations and volume rendering formulas, we demonstrate that our three-dimensional haze rendering approach conforms to the scattering relationship.
- We use a rendering engine based on a three-dimensional scattering formulation to create a simulation dataset for nighttime dehazing. We train our existing network on this dataset and achieve a good nighttime dehazing effect.
- We propose a haze point spread test method based on the optical point spread function to accurately represent haze intensity levels, thereby ensuring that the haze density of the training data is similar to that of the real scene. Based on unique texture relationships of nighttime haze, we propose several network structures and data enhancement methods. Our ablation experiments demonstrate the effectiveness of these improvements.

## 2. Related Work

### 2.1. Image Dehazing

Image dehazing is a form of low-level computer vision image restoration. Tang et al. [5] used a random forest regressor to estimate the degree of haze, randomly sampled from multiple clean images, extracted various multi-scale features related to fog, and then synthesized fog maps. The experimental results once again demonstrated the importance of the dark channel feature DCP [6] and showed that the integration of various features can more accurately estimate the degree of cloud and fog coverage.

Deep learning-based dehazing methods can be divided into two stages: initial network training to obtain intermediate parameters and then substitution of the atmospheric degradation model to calculate the final haze-free image. Recent models tend to directly learn from foggy images to produce haze-free outputs, i.e., end-to-end mapping of the fog image. This approach eliminates the need to solve intermediate parameters, thereby reducing the generation of errors. In 2016, Cai et al. [7] introduced an end-to-end CNN network called DehazeNet. The model takes a contaminated foggy image as input and outputs the transmittance map t(x) of the entire image. The estimated global atmospheric light is then substituted into the degradation model to calculate a clean dehazed image. Ren et al. [8] proposed a multi-scale deep neural network to estimate the transmittance. However, the limitation of these methods is that only the transmittance is estimated separately through the CNN framework, leading to error amplification. To address this issue, Chen et al. [9] proposed a threshold fusion sub-network that utilizes GAN to achieve image dehazing and solves the common problem of unreal ghosting. The latest development in the field of general dehazing is the Dehazeformer, which is based on the transformer structure and has shown promising results. This new approach utilizes the self-attention mechanism of transformers to capture long-range dependencies in the image and effectively restore the haze-free image. Dehazeformer [10] has outperformed several state-of-the-art methods on benchmark datasets, including the RESIDE dataset [11] and the O-HAZE dataset [12].

### 2.2. Nighttime Dehazing

Compared to ordinary image dehazing, image dehazing at night is more challenging due to the complex scene conditions, and research in this area started relatively late. Jing et al. proposed the NDIM algorithm [1], which includes a color correction step after estimating the color characteristics of the incident light. Li et al. [2] distinguished atmospheric light, haze light, glow, and light sources of different colors and proposed the NHRG algorithm based on special processing of glow and recognition of different light sources at night. Ancuti et al. [13] proposed a multi-scale patched pyramid network for artificial light sources to fit the night haze environment. They proposed that the local maximum intensity of each color channel of the night image is mainly contributed by the ambient lighting and introduced the priori of the maximum reflectance, which led to the development of the MRP algorithm [3].

Recently, the team also proposed a new method for constructing foggy data at night called OSFD [4], which is based on scene geometry and involves the two-dimensional simulation of light and object reflectivity. They used the newly generated haze-rendered images to develop a new algorithm and a benchmark test method.

### 2.3. Haze Image Dataset and Rending

The RESIDE dataset [11] is the most well-known image dehazing dataset and is divided into different sets, including the ITS indoor dataset, OTS outdoor dataset, HSTS mixed subjective test set, and SOTS comprehensive subjective test set. Another synthetic dehazing dataset was synthesized by NYU2Depth [14]. The CVPR NTIRE workshop from 2018 to 2021 released a dataset for competition every year, such as the O-HAZE [12] and I-HAZE [15] datasets, which are outdoor and indoor real-shot datasets. Later, outdoor real-shot non-uniform defogging image pair datasets DenseHaze [16] and NH-HAZE [17] were released. However, the amount of these datasets is relatively small, and the scenes are relatively limited, which still leaves a gap in the number of training needs.

The generation of defogging data can be divided into two types: mask construction and physical prior rendering. Mask construction is typically based on the haze atmospheric transmission model, which overlays the haze on the fog-free image, such as the RICE dataset [18]. The other type, such as OSFD [4], divides the scene in the middle of the image semantically and then performs lighting and texture re-rendering based on the physical model.

## 3. Proposed Method

### 3.1. Nighttime Image Haze Model

In nighttime fog and haze scenes, the fog is often not evenly illuminated due to the presence of various complex artificial light sources. Previous studies identified three distinct components of nighttime haze: atmospheric light, glow, and direct light. Atmospheric light is uniformly distributed over the entire image, while glow refers to the halo around a light source. Direct light is the fog that is directly illuminated by light sources of different colors and brightness in the image. Due to various factors, these three types of light may appear differently on nighttime images. Previous studies on dehazing nighttime images processed these components separately, resulting in less-than-optimal results.

To address this issue, we analyzed the principles behind these differences using mathematical formulas. By considering the interactions between the different components, we developed a more effective model for dehazing nighttime images.

The conventional formula for dehazing is given by Equation (1):

(1) I(x)=R(x)∗t(x)+L(x)∗(1−t(x))

where *x* represents the position of the pixel, I(x) represents the signal received by the camera pixel, R(x) represents the signal emitted by the object itself, L(x) represents the atmospheric global illumination, and t(x) represents the transmission rate.

The transmission rate formula is given by Equation (2):

(2) $$t\left(x\right)=e^{-\beta \cdot d(x)},$$

where d(x) represents the distance between the object and the camera at position *x*, β represents the attenuation coefficient, and the exponential term $e^{-\beta d(x)}$ indicates that the attenuation of light due to scattering by the haze is exponentially linear.

The transmission rate formula in Equation (2) incorporates depth information by accounting for the distance between the object and the camera. However, assuming that the light scattering caused by haze only occurs in the depth direction is incorrect. In reality, haze can scatter light in all directions, making it a complex three-dimensional phenomenon. Consequently, the dehazing result obtained from the formula may differ significantly from reality. To account for the complexity of haze and its interaction with light, a more comprehensive approach is needed. This should include the consideration of the concentration of haze in space, the degree of illumination of haze, and its color, among other factors:

(3) $$I\left(x\right)=R\left(x\right)*t\left(x\right)+L\left(x\right)*\left(1-t\left(x\right)\right)+\sum_{k=1}^{n}\hat{S_{k}}\left(x\right)*\sum_{j=0}^{1}\hat{G_{j}}\left(k\right)$$

The term $\sum_{k=1}^{n}\hat{S_{k}}\left(x\right)$ in the formula represents light sources from different directions, while $\sum_{j=0}^{1}\hat{G_{j}}\left(k\right)$ represents scattering in different directions. While this formula provides a rough approximation of the causes of complex haze at night, it is too simplistic to be directly applied to the image processing process. Thus, a more refined and accurate formula is needed to enable its application in practical image processing workflows.

The most essential medium scattering model identifies four primary factors that contribute to the influence of haze on light: absorption, external scattering, emission, and internal scattering. These factors are responsible for image degradation caused by haze.

Absorption refers to the amount of light that is absorbed by haze particles as represented by Equation (4):

(4) $$dL\left(x,\omega \right)/dx=-\sigma_{a}L\left(x,\omega \right),$$

where *x* represents haze particles, while ω represents the angle at which light emerges from the haze particles. The absorption coefficient is denoted by $\sigma_{a}\left(x\right)$, and L(x,ω) represents the intensity of light that emerges from the haze particles at angle ω. Specifically, $\sigma_{a}$ represents the absorption coefficient of the haze particles.

Equations (5) and (6) represent the radiative transfer equation (RTE). In these equations, $-\sigma_{s}L\left(x,\omega \right)$ represents out-scattering, where $\sigma_{s}\left(x\right)$ is the out-scattering coefficient. Emission is represented by $\sigma_{a}L_{e}\left(x,\omega \right)$, while $f_{p}\left(x,\omega ,\omega^{\prime }\right)$ is a phase function. In-scattering is represented by $\int_{s^{2}}f_{p}\left(x,\omega ,\omega^{\prime }\right)L\left(x,\omega^{\prime }\right)d\omega^{\prime }$:

(5) $$\begin{array}{c} dL\left(x,\omega \right)/dx=-\sigma_{t}L\left(x,\omega \right)+\sigma_{a}L_{e}\left(x,\omega \right)\\ +\sigma_{s}\int_{s^{2}}f_{p}\left(x,\omega ,\omega^{\prime }\right)L\left(x,\omega^{\prime }\right)d\omega^{\prime }, \end{array}$$

(6) $$\sigma_{t}\left(x\right)=\sigma_{a}\left(x\right)+\sigma_{s}\left(x\right),$$

When the derivation of the radiative transfer equation is approximated to the volume, the resulting equation is expressed as Equation (7), which is known as the volume rendering equation (VRE) [19]. The VRE is the integral form of the RTE, where *M* represents an opaque surface, and $L_{i}\left(x,\omega \right)$ represents in-scattering in Equation (6):

(7) $$\begin{array}{cc} L(P,\omega )= & T(M)L(M,\omega )\\ & +\int_{x=0}^{d}T\left(x\right)\left[\sigma_{a}\cdot L_{e}\left(x,\omega \right)+\sigma_{s}\cdot L_{i}\left(x,\omega \right)\right]dx, \end{array}$$

here, the transmittance T(x) is the net reduction factor from absorption to out-scattering, which is formulated as follows:

(8) $$T\left(x\right)=e^{-\int_{x}^{p}\sigma_{t}\left(s\right)ds}.$$

The RTE and VRE equations described above are accurate formulas for modeling light scattering in both the real world and computer graphics (CG) renderings. If the goal is to achieve precise image dehazing, it is necessary to abandon the simple dehazing formulas shown in Equations (1) and (2) and instead use the RTE or VRE equations. However, dealing with 3D scattering medium illumination using existing 2D image processing techniques can be challenging, making the problem of fog removal at night quite difficult. Nonetheless, computer graphics rendering can be used to eliminate or generate haze in 3D scattering, providing a potential solution to this problem.

The RTE and VRE equations described above are quite complex and require significant parameter acquisition and calculation overhead. It is difficult to calculate each scattering event based solely on image information. However, we do not actually need to calculate every step of scattering and radiation in detail. Instead, we need to calculate the integral result of scattering or radiation. The intensity of the integral is determined by the light, while the number of levels of integration is determined by the haze particles in the air. The three-dimensional space we ultimately perceive is the result of the final integration. To reconstruct and dehaze the space, we need to approximate multi-level scattering and reach a steady state.

Let us assume that the *n*-level scattered light is represented by $G_{n}$, while the scattering change function for each level of multi-level scattered light is denoted by $f_{ms}$. The formula is as follows:

(9) $$G_{n+1}=G_{n}\cdot f_{ms}.$$

At each level of scattering, the scattered light becomes the light source for the next level of scattering. From a macroscopic perspective, the mutual scattering between light rays is entirely independent. Since the scattering change function $f_{ms}$ is determined by the haze particles and is independent of light intensity, we can perform a multi-level scattering approximation using the following formula:

(10) $$F_{ms}=1+f_{ms}+f_{ms}^{2}+f_{ms}^{3}+\ldots =\frac{1}{1-f_{ms}}.$$

As a result, the scattering steady state $F_{ms}$ will eventually converge to a degree that is linearly related to each level of scattering change function $f_{ms}$ [20].

The above formula indicates that the final result of light scattering must converge to a steady state. In real-world scenes, light is constantly emitted from light sources, while haze particles continuously scatter light. When a camera captures the scene, it integrates the rays over time. As long as the haze and the scene remain unchanged, the resulting image we see remains the same. Intuitively, what we see with the naked eye in the real world is also the light scattered by haze reaching a stable state. Therefore, when we begin to collect haze data pairs in the rendering engine, we do not need to concern ourselves with the complex calculation of light scattering using the VRE process within the engine. Instead, we can use the virtual camera to capture the steady state after calculating the scattering approximation. The resulting data pair automatically conforms to the 3D haze scattering formula.

### 3.2. Construct Nighttime Dehazing Data

**Figure 2:**
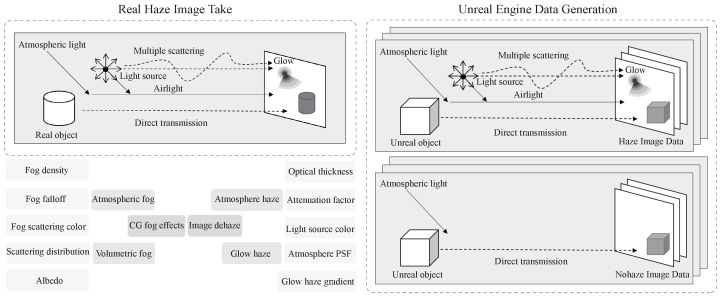
Overview of the experiment framework. Obtain the haze intensity index from the foggy image, select the parameters in the engine, and create a three-dimensional scene to make a paired dataset. Use the network trained on the new dataset to process the foggy image finally. The picture without a dashed box shows the correspondence between image defogging and computer rendering.

Several indicators in computer vision are relevant to the field of image dehazing, such as atmospheric fog and volumetric fog, which correspond to atmospheric haze and glow haze, respectively. Fog density, fog falloff, fog scattering color, scattering distribution, and albedo correspond to the optical thickness, attenuation factor, light source color, atmospheric point spread function (PSF), and glow haze gradient, respectively. Compared to 2D image dehazing, the three-dimensional parameters of computer vision fog effects are more complex, with many parameters that do not have straightforward correspondences, such as cast volumetric shadow and volumetric scattering intensity. A simplified correspondence between these parameters is shown in the lower-left corner of Figure 2.

Creating outdoor real-shot datasets for image dehazing requires the consideration of various factors, such as object movement, changes in lighting conditions, floating fog and haze, and the power of fog machines. Additionally, the distribution of artificial haze and real outdoor haze differs significantly. Therefore, obtaining real nighttime haze and corresponding ground-truth image pairs while maintaining all other conditions is challenging. To address this issue, we propose a method for constructing simulation datasets based on Unreal Engine 5.

Unreal Engine 5 (UE5) [21] is a fifth-generation game engine announced by Epic Games in 2020. To construct our simulation dataset, we used UE5 to generate various night lighting environments. We loaded the project in UE5 and imported environment files containing night haze. Some project and environment files can be downloaded for free from the Epic Store.

To ensure that our simulated dataset closely resembles real-world nighttime lighting environments, we utilized two features of Unreal Engine 5: “Nanite” virtual micro-polygonal geometry and “Lumen” fully dynamic global illumination. Nanite enables us to generate data with full detail and complex textures, while Lumen reacts to scene and lighting changes in real time without requiring specialized ray-tracing hardware. With Lumen, we can render indirect specular and diffuse reflections that bounce infinitely around the scene. These two features ensure that the resulting dataset is highly accurate and closely reflects real-world nighttime lighting environments, with greater rendering accuracy than virtual camera sampling images.

There are two primary types of fog in the engine: exponential height fog and atmospheric fog [22]. They respectively represent the glow and airlight terms in the image dehazing model. The primary difference between night defogging and ordinary defogging is the presence of volumetric fog.

**Figure 3:**
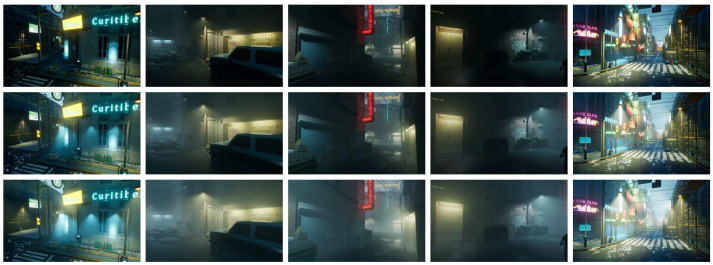
Preview of our simulation dataset. Our dataset is designed to accurately represent the various scattering effects of nighttime haze under artificial light sources. As you move from top to bottom, the smog gradually increases in intensity. In fact, our dataset can be adjusted to include multiple levels of haze intensity, making it an effective tool for modeling a wide range of real-world scenarios.

To generate our simulation dataset, we placed a virtual camera in the rendering engine and moved it through three-dimensional space while fixing it at certain positions. We captured separate images of foggy and non-fog scenes to obtain the paired images, which were saved directly in the project folder. Because the camera was virtual, the resulting images were already in consistent positions, so there was no need for subsequent operations, such as registration. However, it should be noted that some scenes had moving parts and special effects that needed to be removed to maintain consistency across the dataset. We selected various scenes and viewpoints, as shown in Figure 3. In total, we produced 180 pairs of 3000 × 1600 pixel nighttime fog and non-fog image pairs across various scenes.

**Figure 4:**
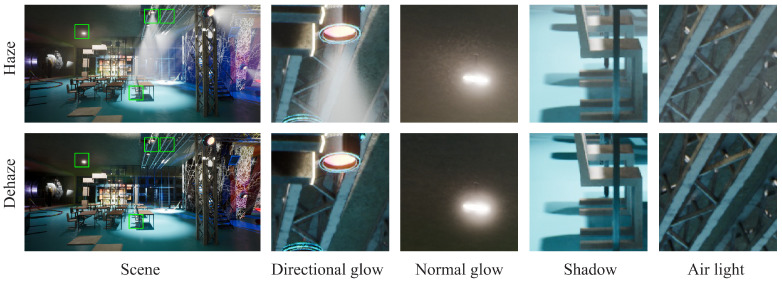
Our generated image haze characteristics comparison. We compare the display of different features in the same picture and highlight the position of each patch on the left.

Upon closer inspection of the collected images, we observed that they exhibit the characteristics of directional haze ${\hat{S}}_{k}\left(x\right)*{\hat{G}}_{j}\left(k\right)$, atmospheric light L(x), and attenuation of the original image signal R(x) (see Figure 4 for details). Furthermore, we observed that the rendered scenes also exhibit a reduction in shadow contrast due to the presence of haze. These characteristic changes are often overlooked in conventional image dehazing. However, with the guidance of the three-dimensional scattering formula, achieving these characteristic changes becomes much easier.

### 3.3. Haze Concentration and Haze Parameters

Existing image dehazing datasets usually only include two classes: hazy and non-hazy. However, in reality, there are various levels of fog, including thin, medium, and dense fog, and different images may have different levels of fog density. The intensity of real-world haze is a continuous value rather than a discrete one. Determining the exact haze concentration in an image can be challenging, which limits the generalizability of single-density fog datasets. An existing dehazing dataset is only suitable for images with similar concentrations of haze, and it may result in insufficient or excessive dehazing for other density haze data.

In an optical system, the distribution of the light field in the output image when the input is a point light source is referred to as the point spread function (PSF). Interestingly, real-world haze also follows this optical principle. When a point light source encounters haze, it forms a large diffuse light field distribution due to scattering. However, when capturing images of haze, various image signal processing operations, such as overexposure correction, dark cutoff, and nonlinear mapping, are often applied, which may introduce nonlinearity [23]. Our method detects HPSFs based on the final image that requires dehazing, without considering any preprocessing operations that may have been performed.

Drawing inspiration from the optical PSF, we propose a new indicator called HPSF (haze point spread function) to analyze the intensity of the haze.

**Figure 5:**
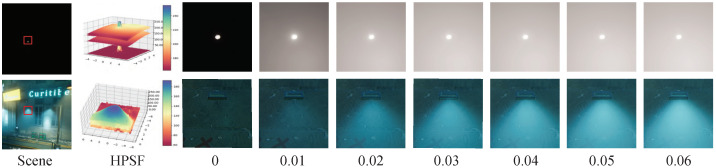
Degradation kernel estimation of haze rendering. The small picture in the upper-left corner illustrates the relationship between haze intensities and the actual haze point spread function (HPSF). The haze intensity increases gradually from left to right (0–0.06). The upper images show the haze degradation of a point light source, while the lower images show the haze change of a directional light source in a simulated scene. The lower images were taken from the red box in the left scene.

Point light through a lens without aberration should be an impulse function signal. Like the optical PSF, we assume that objects in the scene without haze should maintain consistently smooth or step signals, as shown in the first and second rows in Figure 5.

The acquisition and calibration of our different HPSFs are entirely consistent with the optical acquisition and calibration of the PSF. We verify the point spread function of the image and the changes at the edges of the image. For specific operational steps, please refer to our previous work [24] on calibration testing methods.

By analyzing the HPSF of a specific area in the image, we can determine the corresponding haze density through a predefined relationship as illustrated by the numbers in Figure 5. This analysis method can measure the density of fog effects, such as exponential height fog or volume fog density in render engines. The haze density obtained from this metric corresponds to different haze concentration datasets and dehazing networks trained on various concentration datasets.

Therefore, we can generate customized data pairs suitable for the intensity of haze present in the image. To simplify usage, we can create haze data pairs with different discrete intensities in advance, which can save the time required to generate images in the engine. When processing images with varying haze intensities, we only need to load the corresponding trained network checkpoint.

### 3.4. Improved Night Image Dehazing

We selected SADNet [25], which represents a small network based on the UNet improvement, and Restormer, with a more complex network and better fitting abilities, as the night defogging network.

For the network, we propose two improvements, preprocessing improvement and loss improvement, both of which are specially designed based on the unique characteristics of night haze scenes.

**Figure 6:**
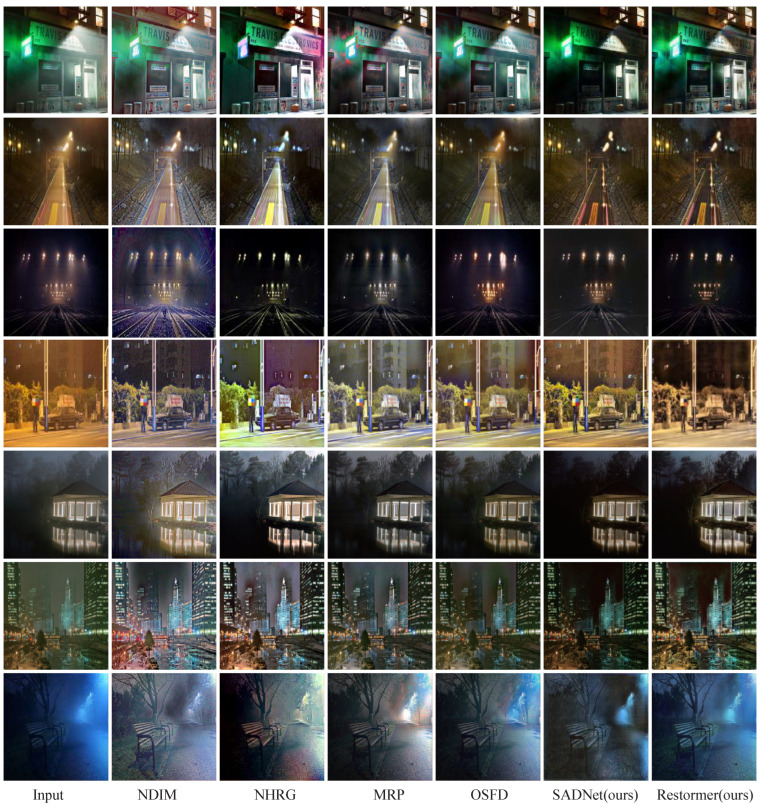
Image dehazing result comparison. Comparison with other nighttime dehazing methods, the compared dataset is based on flicker dataset [3].

We made improvements to the data preprocessing step. Most neural networks preprocess images when inputting them to the network by applying data augmentation operations, such as cropping, horizontal flipping, and vertical flipping. However, after observing both the simulated dataset in Figure 3 and the real-time non-reference dataset in Figure 6, we noticed that there is a certain distribution relationship between directional irradiation. For instance, nighttime fog tends to have a distribution from top to bottom, with the smaller part at the top and larger part at the bottom. We also observed a statistical law in the distribution of different light source directions.

Therefore, we modified the existing data augmentation method by only allowing horizontal enhancements and random cropping, while not allowing vertical data augmentation. It is worth noting that this does not imply that the network and data are unable to handle non-uniform haze in other directions. Instead, it is to keep the dataset in line with the distribution law of the real scene and achieve better training results.

Next, we improved the structure of the loss used in both the SADNet and Restormer structures. Previously, these networks used a simple L1 loss, which is well suited for global image processing tasks and allows the network to uniformly learn overall feature changes in the image. However, for nighttime fog removal, the network needs to learn more local features. In Figure 4 and Figure 5, it can be seen that there is often a large amount of haze around the brightest light source area in the image, which is the most challenging part of nighttime haze removal.

To address this issue, we propose a modified loss function that places more emphasis on the local features in the image. The simple L1 and L2 loss formulas are as follows:

(11) $$L_{l1}=I_{out}-I_{gt}$$

(12) $$L_{l2}={\left(I_{out}-I_{gt}\right)}^{2}$$

We improved the loss formula to assign greater weight to the brighter parts of the image, which is essential for effective nighttime fog removal. During the training process, the loss of each small image in every batch is calculated separately, and the total loss is then recalculated. The formula for our newly proposed loss function is called ’light loss’ and is as follows:

(13) $$L_{Light}=I_{out}*\left(I_{out}-I_{gt}\right)$$

## 4. Experimental Results

To evaluate our technique, we conducted a series of comprehensive experiments. Specifically, we conducted detailed comparative experiments to investigate the effects of different HPSFs, losses, and data preprocessing methods, and demonstrated their effectiveness. Additionally, we compared our technique with other methods on both simulation and real datasets.

### 4.1. Ablation Study with Different HPSF Concentrations

We conducted comparative experiments to demonstrate the advantages of using a network that corresponds to the HPSF concentration data. Our results indicate that a network trained on a similar density dataset produces better haze removal results. As shown in Table 1, the best results for fog removal from 0.1 to 0.4 (test) were achieved using the network trained on the same haze concentration dataset. The networks trained on other concentrations produced comparatively poorer results.

### 4.2. Ablation Study with Data Preprocessing

In order to demonstrate the effectiveness of our improved data preprocessing methods, we conducted ablation experiments on our haze dataset. We aggregated all concentration datasets for testing purposes. The results are shown in Table 2. Our experiments demonstrated that horizontal flipping can effectively increase diversity and improve the dehazing performance. However, flipping the images up and down resulted in incorrect diversity and decreased the final outcome of the dehazing process.

### 4.3. Ablation Study with Loss

We propose a novel loss function based on the distribution of nighttime haze. Our ablation experiments demonstrate the effectiveness of our proposed approach. As presented in Table 3, we compared our loss function with the traditional loss function at varying levels of haze concentration (0.1–0.6). Our results indicate that our loss function is more effective at higher levels of haze, and shows a stronger ability to suppress the presence of haze in the vicinity of light sources.

### 4.4. Testing on Synthetic Images

To demonstrate the superiority of our proposed method, we compared it with several existing nighttime dehazing methods, including NDIM [1], NHRG [2], MRP [3], and OSFD [4].

We observed that dehazing night images is a challenging task, and some methods fail to surpass the input hazy images in terms of peak signal-to-noise ratio (PSNR) [26] and structure similarity index measure (SSIM) [27]. However, our proposed method significantly outperforms the other methods in terms of PSNR, SSIM and CIE2000 [28]. The computation time is also comparable to the fastest method. The PSNR and SSIM are metrics where a larger value indicates better performance, while CIE2000 and computation time are metrics where a smaller value indicates better performance. In some of the charts, we use the symbol ↑ to represent that a larger value is better, and ↓ to represent that a smaller value is better. All evaluation methods are based on functions that come with Python. All objective evaluation indicators are compared as shown in Table 4.

Qualitatively, NDIM suffers from severe global color casts and cannot distinguish between the brightening of light and the brightening caused by haze scattering. NHRG and MRP address these issues to some extent, but they still exhibit significant local color cast defects. OSFD produces good color and performs well on uniform haze removal due to its large training dataset, but it is not effective enough in removing non-uniform haze. In contrast, our proposed method performs better on simulated datasets but cannot completely remove glare caused by fog. Please refer to the first row of Figure 6 for detailed comparisons.

### 4.5. Evaluation on Real Photographs

We utilized the Flicker dataset [3], which is commonly used for subjective comparisons in the field of nighttime dehazing, as our real-shot dataset. Our approach of obtaining haze scattering based on 3D rendering was found to be highly effective. Our results effectively suppress the surrounding haze observed from artificial light sources, preserving the shape and brightness of the light source while removing the surrounding haze as shown in the second and third rows of Figure 6. Our method also produces very few color casts. For scenes with blue and yellow haze light sources, we maintain the lighting color of the environment and remove the colored haze as demonstrated in the fourth and seventh row of Figure 6.

Additionally, our method performs well on uniformly hazy night scenes, maintaining the details of the bright and dark parts while removing the haze feeling in the space. As shown in the comparison between the fifth and sixth rows of Figure 6, our buildings are clearer.

Overall, our method has a better understanding of the various scattering laws of haze due to the dataset learned from the three-dimensional scattering formula. Our approach is more capable of handling complex haze and light relationships and is better suited for processing subjective real-shot datasets, producing better colors and texture details that are more in line with real-world haze scenes.

## 5. Conclusions

Real night dehazing mechanisms are complex, and datasets are difficult to capture. In this regard, we proposed several innovations:

- We propose a new compositing method that leverages rendering techniques to recreate complex real-world lighting and haze. Compared to other data construction methods, our results are more consistent with real scenes and optical scattering models.
- To match haze images of different concentrations and render datasets at different concentrations, we propose a haze point spread function (HPSF) using the light point spread function (PSF) method.
- We improved the data preprocessing method and loss function of the neural network based on the image characteristics of nighttime haze images.

We conducted various experiments to demonstrate the effectiveness of these improvements. Our dehazing results achieved good performance on both simulated and real-world data. Overall, our proposed method provides a more realistic way to generate nighttime dehazing datasets, and the improvements we made to the network structure and training process led to better performance. These findings have important implications for the future development of more efficient dehazing technologies.

## Figures and Tables

**Table 1:** Objective results on different concentration test sets after training on different concentration datasets.

Train Concentration	0.1	0.2	0.3	0.4
test 0.1 (PSNR↑/SSIM↑)	**30.94**/**0.956**	29.58/0.955	28.31/0.950	28.23/0.933
test 0.2 (PSNR↑/SSIM↑)	27.64/0.932	**28.59**/**0.937**	28.03/0.928	26.84/0.920
test 0.3 (PSNR↑/SSIM↑)	23.97/0.895	25.65/0.903	**27.16**/**0.915**	26.48/0.912
test 0.4 (PSNR↑/SSIM↑)	20.10/0.845	20.23/0.852	21.69/0.861	**26.02**/**0.905**

**Table 2:** Quantitative comparison of different data preprocessing methods on simulation datasets.

Horizontal Flip		✓		✓
Vertical Flip			✓	✓
PSNR↑	29.17 (2%)	**29.37** (0%)	29.02 (4%)	28.91 (5%)
SSIM↑	0.860 (6%)	**0.869** (0%)	0.862 (5%)	0.864 (4%)

**Table 3:** Quantitative comparison of different losses on different concentrations simulation datasets.

Haze Concentration	0.1	0.2	0.3	0.4	0.5	0.6
L1 Loss (PSNR/SSIM)	30.59/0.956	27.54/0.933	23.68/0.890	21.43/0.851	19.04/0.796	17.80/0.757
LightLoss (PSNR/SSIM)	30.94/0.956	28.59/0.937	27.16/0.915	26.02/0.905	25.23/0.861	24.89/0.852

**Table 4:** Qualitative results of various nighttime dehazing methods on simulated datasets.

Method	Input	NDIM	NHRG	MRP	OSFD	SADNet (Ours)	Restormer (Ours)
PSNR↑	26.17 (44%)	20.56 (176%)	23.95 (87%)	26.43 (40%)	27.72 (21%)	28.44 (11%)	**29.37** (0%)
SSIM↑	0.737 (101%)	0.680 (144%)	0.754 (88%)	0.792 (59%)	0.837 (24%)	0.854 (11%)	**0.869** (0%)
CIE2000↓	146.3 (42%)	130.1 (26%)	118.7 (15%)	132.6 (28%)	128.9 (25%)	103.3 (0%)	**102.8** (0%)
Time/s↓		20.58	25.03	1.56	**0.772**	1.692	1.988

## Data Availability

The provided link leads to a GitHub repository https://github.com/madone7/Nighttime-Image-Dehazing-by-Render, (accessed on 10 July 2023).
